# A Case–Control Study Supports Genetic Contribution of the *PON* Gene Family in Obesity and Metabolic Dysfunction Associated Steatotic Liver Disease

**DOI:** 10.3390/antiox13091051

**Published:** 2024-08-29

**Authors:** Evelien Van Dijck, Sara Diels, Erik Fransen, Tycho Canter Cremers, An Verrijken, Eveline Dirinck, Alexander Hoischen, Geert Vandeweyer, Wim Vanden Berghe, Luc Van Gaal, Sven Francque, Wim Van Hul

**Affiliations:** 1 Centre of Medical Genetics, University of Antwerp and Antwerp University Hospital, 2650 Edegem, Belgium; 2 Department of Endocrinology, Diabetology and Metabolic Diseases, Antwerp University Hospital, 2650 Edegem, Belgium; 3 Laboratory for Experimental Medicine and Paediatrics, Translational Sciences in Inflammation and Immunology, University of Antwerp, 2610 Wilrijk, Belgium; 4 Department of Human Genetics and Department of Internal Medicine, Radboud University Medical Center, 6525 GA Nijmegen, The Netherlands; 5 Cell Death Signaling–Epigenetics Lab, Department Biomedical Sciences, University of Antwerp, 2610 Wilrijk, Belgium; 6 Department of Gastroenterology and Hepatology, Antwerp University Hospital, 2650 Edegem, Belgium

**Keywords:** obesity, metabolic dysfunction associated steatotic liver disease (MASLD), paraoxonase (PON), genetics, antioxidants, oxidative stress

## Abstract

The paraoxonase (*PON*) gene family (including PON1, PON2, and PON3), is known for its anti-oxidative and anti-inflammatory properties, protecting against metabolic diseases such as obesity and metabolic dysfunction-associated steatotic liver disease (MASLD). In this study, the influence of common and rare *PON* variants on both conditions was investigated. A total of 507 healthy weight individuals and 744 patients with obesity including 433 with histological liver assessment, were sequenced with single-molecule molecular inversion probes (smMIPs), allowing the identification of genetic contributions to obesity and MASLD-related liver features. Polymorphisms rs705379 and rs854552 in the *PON1* gene displayed significant association with MASLD stage and fibrosis, respectively. Additionally, rare *PON1* variants were strongly associated with obesity. This study thereby reinforces the genetic foundation of *PON1* in obesity and various MASLD-related liver features, by extending previous findings from common variants to include rare variants. Additionally, rare and very rare variants in *PON2* were discovered to be associated with MASLD-related hepatic fibrosis. Notably, we are the first to report an association between naturally occurring rare *PON2* variants and MASLD-related liver fibrosis. Considering the critical role of liver fibrosis in MASLD outcome, PON2 emerges as a possible candidate for future research endeavors including exploration of biomarker potential.

## 1. Introduction

A staggering one-third of the population is currently affected with metabolic dysfunction-associated steatotic liver disease (MASLD) [1]. This hepatic manifestation of metabolic syndrome is characterized by lipid accumulation surpassing 5% of hepatocytes, combined with at least one metabolic dysregulation factor [2]. MASLD encompasses a spectrum of liver disease stages, initially confined to hepatic steatosis, but potentially progressing to more severe stages of hepatocellular injury, known as metabolic dysfunction-associated steatohepatitis (MASH). MASH is marked by the additional presence of liver inflammation, hepatocellular ballooning, and potentially liver fibrosis alongside hepatic steatosis. Furthermore, MASH patients have an increased risk of progression to liver cirrhosis and ultimately hepatocellular carcinoma [3]. A steep rise in incidence has propelled MASLD to become the leading cause of liver transplantation, highlighting the urgent need for enhanced disease prevention and treatment strategies [1]. However, the pathogenesis of MASLD is complex, including genetic, epigenetic, environmental, and clinical factors, hampering its complete elucidation.

Obesity, defined as an excessive fat accumulation that presents a health risk, is one of the most important risk factors for MASLD development [4]. Obesity induces chronic low-grade inflammation and increased oxidative stress in the body. These processes are strongly interconnected, and not only play a role in the progression of many diseases, including MASLD, but they are also independently capable of inducing both obesity and MASLD, as reviewed elsewhere [5,6,7,8]. Oxidative stress originates from an imbalance between the generation of reactive oxygen species (ROS) and defense against these through antioxidant mechanisms. This imbalance can arise at the genetic level, with variants in key antioxidant genes affecting protein function and concomitantly leading to dysregulation of normal oxidative balance, thereby propelling individuals toward MASLD and obesity development [9,10].

For this reason, our attention is directed towards a specific family of anti-inflammatory and antioxidant enzymes, called the paraoxonase (*PON*) gene family. This family contains three members, PON1, PON2, and PON3, sharing significant levels of amino acid homology. *PON1* and *PON3* are mainly expressed in liver hepatocytes, associate with high-density lipoprotein in the circulation, and prevent peroxidation of low-density lipoprotein [11,12,13]. Some expression is also found in cholangiocytes, albeit significantly less. In contrast, *PON2* exhibits a more widespread expression, showing expression in almost all liver cell types, with the highest expression in both hepatocytes and cholangiocytes [14,15]. Unlike PON1 and PON3, PON2 is confined to intracellular compartments, predominantly localizing to the mitochondria and the endoplasmic reticulum. Here, PON2 mitigates oxidative damage, thereby playing an important role in pathophysiological mechanisms associated with MASLD, such as mitochondrial dysfunction and endoplasmic reticulum stress [7,16,17,18]. 

As a result of their antioxidant and anti-inflammatory properties, PON proteins have been implicated in various metabolic diseases [19,20,21]. Specifically, their involvement in liver lipid metabolism and liver-related oxidative stress highlights their significance in the etiology and progression of obesity and MASLD. Among the PON family, PON1 shows the strongest association with metabolic disorders, functioning as a key regulator of glucose and lipid homeostasis [22]. Alongside reports from genetically altered animal and cellular models, natural human variations in *PON* expression and activity have also been associated with metabolic features [23]. The variability in enzyme level and catalytic activity of the PON proteins is (partly) affected by known single nucleotide polymorphisms (SNPs) [24,25]. However, these SNPs only explain part of the variability, driving research into new directions, such as the study of rare variants [26]. In this context, a study on PON1 has supported this approach by identifying rare variants that predict PON1 activity, underscoring the potential importance of exploring these less-studied genetic variants [27].

In conclusion, genetic variants in antioxidant genes may play an important role in the initiation and exacerbation of both obesity and MASLD. This study therefore aims to further substantiate our understanding of the involvement of the anti-oxidant *PON* genes in the genetic framework of obesity and MASLD. To broaden our exploration for genetic contribution, we conducted a targeted genetic screening encompassing not only well-known common polymorphisms but also less-studied rare variants. Additionally, our extensively characterized patient cohort enables us to pinpoint possible genetic contributions to either obesity or specific MASLD-related liver hallmarks such as hepatic steatosis, hepatocellular ballooning, lobular inflammation, fibrosis stage, and general MASLD disease progression. We report an association between common *PON1* SNPs and MASLD severity and fibrosis. Additionally, analysis of rare variants reveals an association with obesity for *PON1* and MASLD-related liver fibrosis for *PON2*.

## 2. Materials and Methods

### 2.1. Study Populations

A cohort of 744 unrelated Caucasian patients living with obesity (body mass index (BMI) ≥ 30 kg/m^2^) with(out) associated MASLD was recruited from the obesity clinic of the Antwerp University Hospital. All patients underwent extensive metabolic profiling [28]. A cohort of 507 lean and unrelated Caucasian individuals (18.5 kg/m^2^ ≤ BMI < 25 kg/m^2^) was recruited among employees from the University of Antwerp, the Antwerp University Hospital, and among couples seeking prenatal counseling at the Centre for Medical Genetics in Antwerp (due to high maternal age or increased triple test). Basic characteristics of both cohorts are displayed in Table 1. Extensive baseline characteristics relating to metabolic and liver health of obese patients are displayed in Table 2. Informed consent was obtained for all individuals involved. The study was approved by the Ethics Committee of the Antwerp University Hospital (references A04-21 and 6/25/125, Belgian registration number B30020071389), and approval was given according to the declaration of Helsinki. 

### 2.2. Weight and Length Assessment

Weight and length were measured in all cases and controls. For cases, weight was measured with a digital scale to 0.2 kg and height was measured to 0.5 cm. For lean controls, weight and length were measured to 1 kg and 1 cm. Subsequently, BMI was calculated by dividing a person’s weight (in kg) by their squared height (in m^2^).

### 2.3. Liver Assessment

From the original cohort of 744 unrelated patients with obesity, a subset of 433 patients underwent liver biopsy. The procedure was performed either percutaneously (16G Menghini), transjugularly (16G transjugular liver biopsy needle), or perioperatively (16G Trucut needle) after informed consent. Hematoxylin–eosin, Sirius red, reticulin, and Perl’s iron stains were performed on paraffin-embedded liver slides. Subsequently, blind assessment of liver biopsies was conducted by two experienced pathologists. The NASH Clinical Research Network (NASH-CRN) scoring system was used to assess steatosis grade (S: 0–3), lobular inflammation (I: 0–3), hepatocellular ballooning (B: 0–3), and fibrosis stage (F: 0–4) [29,30]. Additionally, using these parameters, the MASLD stage parameter was introduced to subclassify the patient cohort into four relevant disease stages: (i) no MASLD, (ii) isolated hepatic steatosis, (iii) MASH without or with mild fibrosis, and (iv) MASH with moderate to severe fibrosis (Supplementary Table S1). Only patients that had no or very low alcohol consumption (<20 g/day), no previous weight loss surgery, and no other etiology of liver disease such as viral hepatitis or autoimmune disease, were included.

### 2.4. smMIPs Sequencing

**Figure 1:**
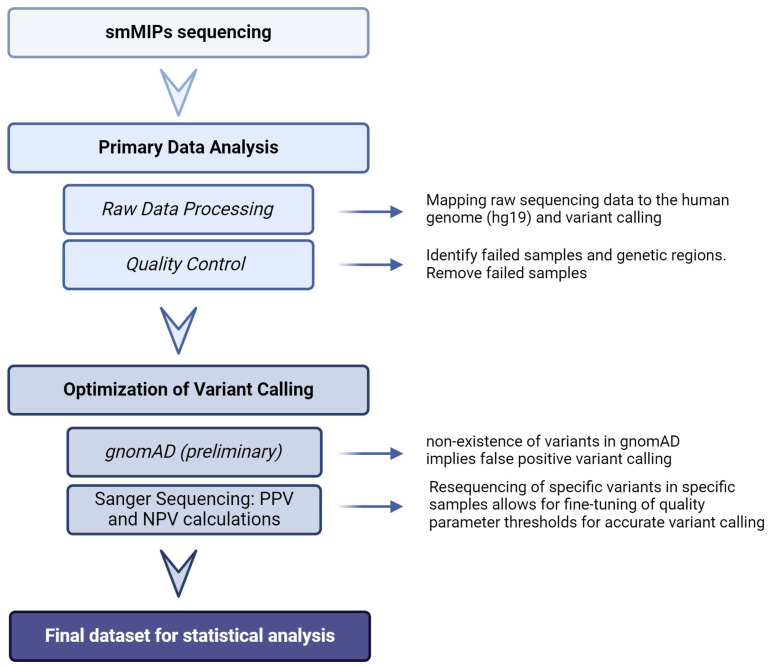
smMIPs data analysis flowchart. The flowchart visualizes the main steps in data processing including primary data analysis (raw data analysis and quality control), optimization of variant calling through gnomAD and Sanger sequencing, respectively, and finally, statistical analysis. PPV = positive predictive value, NPV = negative predictive value. Created with BioRender.com (accessed on 20 August 2024).

Single-molecule molecular inversion probes (smMIPs), a targeted next-generation sequencing (NGS) approach, were applied for the enrichment of *PON1*, *PON2*, and *PON3.* For this purpose, DNA was extracted from whole blood samples. Double-tiled smMIPs (IDT, Leuven, Belgium) were designed to cover all *PON* exons, exon–intron boundaries (with a minimum of 10 base pairs overhang), and limited 5′UTR and 3′UTR regions (genome build GRCh37/hg19) using the MIPgen pipeline [31]. The presence of common variants was taken into account in the design. In total 55, 52, and 46 probes were selected to cover *PON1*, *PON2,* and *PON3,* respectively. The protocols of O’Roak et al. [32] and Dutta et al. [33] were followed for sample preparation. Briefly, probes were pooled together and phosphorylated. Hybridization of smMIPs to their target regions followed by gap-filling and ligation resulted in circularized DNA molecules while remaining linear probes and genomic DNA were digested by exonuclease treatment. Polymerase chain reaction was performed to amplify captured regions and barcode samples. Lastly, a purification step was carried out before sequencing. In total three 150 cycles of NextSeq500/550 Mid Output kits (Illumina, San Diego, CA, USA) were loaded with 1.7 pmol samples for sequencing in a dual indexed, 2 × 76 bp format. A comprehensive overview of the downstream data analysis is presented in Figure 1. 

### 2.5. Primary Data Analysis

#### 2.5.1. Raw Data Processing

Data processing was performed using an in-house bioinformatics pipeline consisting of (i) read trimming, (ii) aligning reads from fastq data to the human reference genome (hg19) using Burrows–Wheeler aligner (v0.7.4) [34], (iii) filtering of non-exact aligned reads and (iv) multi-sample variant calling using the Unified Genotyper from GATK (v3.5.0) [35]. Minimum base quality was set to 30 and all data with a mapping quality below 50 (corresponding to a mapping accuracy of 99.999%) were discarded. 

#### 2.5.2. Quality Control

The coverage quality of the data was assessed threefold: (i) the mean coverage per sample across all smMIPs, (ii) the median coverage per transcript, and (iii) the percentage of each transcript reaching a minimum coverage of 5× with 95% confidence intervals (Supplementary Table S2). Failed samples were removed. No failed regions were identified. Data from three runs were merged for further processing.

### 2.6. Optimization of Variant Calling

#### 2.6.1. GnomAD

Preliminary filtering of the NGS data involved dividing the dataset according to variant presence in the Genome Aggregation Database (gnomAD v4.0.0 [June 2020]) [36]. Variants absent from the gnomAD database were assumed to be false positives. Initial values for quality parameters “quality by depth”, “total allele depth”, and “allelic ratio” (number of alternative alleles/total number of alleles) were established as a preliminary step toward optimizing variant calling.

#### 2.6.2. Sanger Sequencing

Sanger sequencing was conducted on 85 samples to refine quality parameter thresholds and ensure accurate final variant calling. Primers were designed using Primer3Plus [37]. Upon request, primer sequences can be made available. Briefly, target amplification was performed by GoTaq DNA polymerase-mediated PCR (Promega Corporation, Madison, WI, USA). The mixture was then treated by exonuclease I (New England Biolabs, Ipswich, MA, USA) and calf intestine alkaline phosphatase (CIAP, Roche Applied Science, Hoffmann–La Roche AG, Basel, Switzerland) at 37 °C to remove excess primer and unincorporated dNTPs. Finally, sequencing was performed using ABI BigDye Terminator v1.1 Cycle Sequencing kits (Thermo Fisher Scientific, Waltham, MA, USA) on an ABI Prism Genetic Analyzer 3130xl (Applied Biosystems Inc., Foster City, CA, USA). The samples selected for Sanger sequencing were dispersed across cohorts, genes, and regions within these genes, with quality parameter values similar to those established by prior gnomAD-based filtering. For a detailed overview of the Sanger-based variant calling optimization, see Supplementary Data: PPV and NPV calculations.

### 2.7. Statistical Analysis

All statistical association tests were conducted using the Variant Association Tools (vTools) package (v2.7.0) [38]. Tests were conducted at two variant levels: single variant tests for common variants with relatively high minor allele frequencies (MAF) and gene-based tests for rare variants. Because the presence of obesity and consequently MASLD is high in the general population, MAFs in gnomAD are less reliable as a reference. Therefore, common, rare, and very rare variants are defined as variants with a minor allele frequency of >5%, <1%, and <0.12% in our lean population cohort, respectively. The rationale behind the 0.12% is that our control population is too small to support an allele frequency of 0.1%. However, we can substantiate a 1/900 fraction, which corresponds to a 0.12% frequency. Association for single variants is tested through Fisher’s exact test. To account for multiple testing, q-values were calculated with the R-package qvalue (version 2.4.2) using the method of Storey and Tibshirani [39]. This controls the false discovery rate (FDR), with the q-value describing how likely an identified association (or significant *p*-value) represents a false positive. In addition, the enrichment of low *p*-values is visualized through a Q-Q plot, where the distribution of the observed *p*-values is plotted against the uniform distribution (U(0,1)), which is the expected null distribution, in case no genes are associated with the phenotype. An enrichment of significant *p*-values compared to the null distribution indicates the presence of true positive associations. As recommended by Lee et al. [40], gene-based testing is conducted for both rare and very rare variants under a variety of models including the adaptive burden tests “Variable Threshold” (VT) [41], and “Kernel-based Adaptive Cluster” (KBAC) [42], the omnibus test “Fisher” [43] and the variance component test “C-alpha” [44]. 

## 3. Results

### 3.1. Genetic Variant Calling

Three NGS sequencing runs were conducted, yielding 88.6%, 88.6%, and 86.8% sequencing data with a minimum base calling quality higher than 30. Samples with a mean coverage ≤ 10 were excluded from the analysis, resulting in a total of 501 controls and 741 patients retained for analysis. The patient cohort achieved an average median coverage of 172×, with an average of 99.4% of transcripts reaching a 5× sequencing depth across all genes. Similarly, the control cohort exhibited an average median coverage of 289×, with an average of 99.4% of the transcripts reaching a 5× sequencing depth across all genes (Supplementary Table S2). This demonstrates high sequencing quality in both cohorts, with no missing regions reported. Cohort-specific coverage analysis confirmed at least equally robust coverage in the control cohort, minimizing the risk of false positive results due to sequencing quality variations. To accurately identify variants, a minimum average coverage (or total allele depth) threshold of five was established based on Sanger resequencing of 85 samples, including a total of 109 variants. Likewise, quality parameter thresholds were set for quality by depth (>3.89) and allelic ratio (≥0.2 and ≥0.8 for heterozygous and homozygous variants respectively). A total of 100 unique genetic variants (Supplementary Table S3), were retained for further statistical analysis. A detailed overview of the applied filtering is available in the supplementary data: PPV and NPV calculations.

### 3.2. Single Variant Association Tests Show No Associations with Obesity

**Figure 2:**
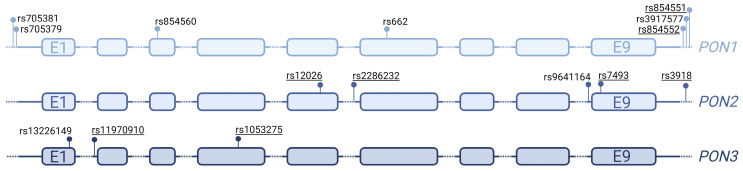
Visual representation of common variants in *PON1*, *PON2*, and *PON3*. Variants are indicated by SNP IDs. Variants in linkage disequilibrium are underlined. E1 and E9 represent exon 1 and exon 9, respectively. Created with BioRender.com (accessed on 20 August 2024).

A total of 15 common variants were discovered in the three *PON* genes: seven in *PON1*, five in *PON2*, and three in *PON3* (Table 3, Figure 2). After linkage disequilibrium (LD) correction, six *PON1*, two *PON2*, and two *PON3* independent common variants remain. To evaluate the association of these common variants with obesity, MAFs were individually compared between the cohort with obesity and the healthy weight control cohort. Association was tested using Fisher’s exact test followed by FDR to account for multiple tests. No significant associations were observed (Table 3).

### 3.3. Gene-Based Association Tests Indicate an Association between Rare PON1 Variants and Obesity

In addition to common variants, the smMIPs pipeline also captures rare variants. A total of 79 rare variants of which 61 very rare variants were identified across the three *PON* genes, as detailed in Table 4. Gene-based tests are conducted for these rare variants, evaluating the cumulative impact of all (very) rare variants within each gene collectively, rather than analyzing them individually. This approach circumvents the need for extremely large sample sizes required to achieve sufficient statistical power for testing individual rare variants. Given the unknown genetic architecture underlying the disease, with variants that can have either deleterious or beneficial effects on disease development and/or progression, associations are tested under a variety of models. These include two adaptive burden tests (VT and KBAC), a variance component test (C-alpha), and an omnibus or combined test (Fisher). Ideally, statistical significance is achieved across all models, indicating a robust effect [40]. When comparing individuals with obesity to lean controls, significant results were obtained for the *PON1* gene. For rare variants, all test models demonstrated significance, whereas for very rare variants, all tests but the C-alpha test were significant (Table 5).

### 3.4. Single Variant Association Tests Indicate an Association between PON1 Common Variants and Several MASLD-Related Features

**Figure 3:**
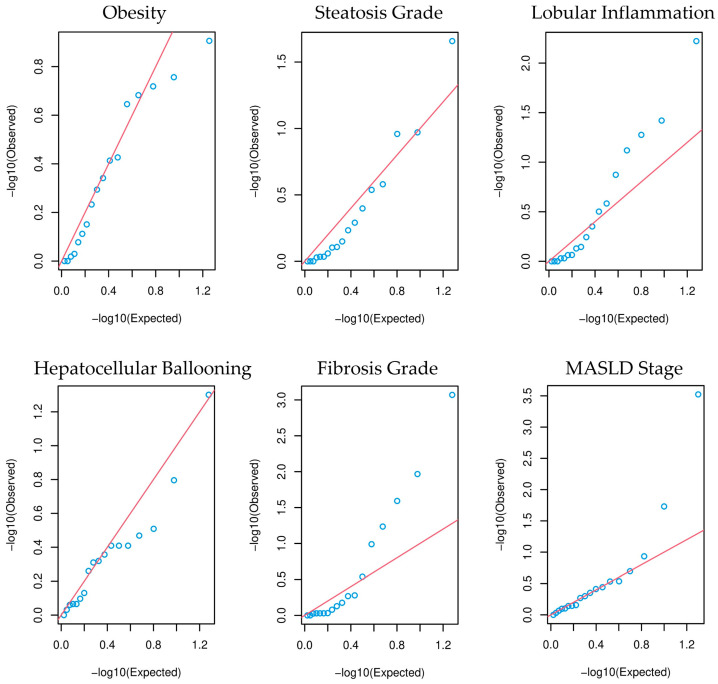
Q-Q plots per testing group. The Q-Q plot visualizes the enrichment of low *p*-values compared to the null distribution U(0,1), for the single variant Fisher’s exact test. The red line is the uniform distribution (U(0,1)). Each blue dot represents a single common variant.

For the 433 patients who underwent additional liver biopsy, we conducted tests to evaluate the association of common variants with specific hallmarks of MASLD. These include (i) steatosis grade, (ii) lobular inflammation, (iii) hepatocellular ballooning, (iv) fibrosis stage and their combined (v) MASLD stage. Each common variant is individually tested for association with each feature using Fisher’s exact test followed by FDR analysis. For these five separate association analyses, patients were split into two groups based on the presence or absence of either hepatic steatosis, lobular inflammation, hepatocellular ballooning, or fibrosis. Additionally, individuals with no MASLD or only hepatic steatosis were compared to those with MASH and different stages of liver fibrosis. The results of all tests are summarized in Table 3. Remarkably, all nominally significant associations were observed in the *PON1* gene, with at least one nominally significant result per testing group. Following multiple testing corrections by FDR, only two variant associations retained a q-value below 0.05: a *PON1* 3′UTR variant (rs854552) for fibrosis and a *PON1* 5′UTR (rs705379) variant for the MASLD stage. Both variants exhibit enrichment in the control cohorts, having no fibrosis and no MASLD or hepatic steatosis compared to varying stages of fibrosis and MASH with varying stages of fibrosis, respectively (Supplementary Table S4). Although no other associations remained significant upon FDR analysis, the Q-Q plots (Figure 3) reveal that a common variant in the lobular inflammation group (rs705379), and to a lesser extent a variant in the steatosis grade group (rs3917577), exhibit visual enrichment of low *p*-values compared to a uniform distribution.

### 3.5. Gene-Based Association Tests Indicate an Association between Rare PON2 Variants and MASLD-Related Liver Fibrosis

To examine the association between (very) rare *PON* variants and MASLD-related liver features, the same five groups are evaluated. Again, the cumulative effect of all (very) rare variants within each gene is evaluated for association with each group. An overview is provided in Table 4. Highly significant results were consistently observed for rare and very rare *PON2* variants when comparing samples without liver fibrosis to samples with varying stages of liver fibrosis. Some borderline significant results were obtained for rare *PON3* variants when comparing patients without inflammation or fibrosis to those with varying stages of these liver injury parameters. These were, however, not consistently significant across all tests and were never significant for very rare variants, indicating limited evidence for a true association.

## 4. Discussion

In this study, smMIPs sequencing was used to target coding and UTR regions of three antioxidant *PON* genes in patients with obesity with(out) associated MASLD and lean control individuals. We aimed to discover common and rare variants contributing to obesity and/or MASLD pathogenesis. A total of 100 variants, including 15 common variants were identified across the *PON* genes. After adjusting for LD, a total of eight *PON1*, two *PON2*, and two *PON3* independent common variants remained. For each detected common variant, MAFs of our control cohorts were similar to those found in GnomAD for European non-Finnish ethnicities (Supplementary Table S4). A total of 79 rare, including 61 very rare, variants were additionally identified for gene-based association analysis.

No significant associations were found between common *PON* variants and obesity. For *PON1* specifically, there have, however, been reports showing an association between *PON1* SNPs and obesity [45,46,47]. Our lack of association might be related to discrepancies in ethnicity, the targeted regions (as some associated SNPs were reported to be intronic), or our limited cohort size. Although several reports have described an association between PON3 and body weight, supported by studies in mice [48,49], our findings align with current genetic knowledge, which shows no associations between common *PON3* variants and obesity or related traits. This absence of significant association might be attributable to the nature of the identified SNPs: two SNPs are synonymous and therefore do not impact overall protein structure, while the third is intronic, with an unknown impact on protein function. For *PON2*, there have not been consistent associations to either obesity or body weight features in *PON2* knockout mice [50,51], which is reflected by our study’s lack of genetic association.

Gene-based association analysis identified associations between rare and very rare *PON1* variants and obesity across all but one test, indicating a robust association in our cohort. The only test not reaching statistical significance was the C-alpha test for very rare variants. C-alpha is a variance component test, meaning that, unlike conventional burden tests, it considers the possibility of beneficial variants rather than only assuming deleterious effects. A possible explanation for the lack of significance for this test specifically could therefore be that most very rare *PON1* variants in our data are deleterious. Overall, these results strengthen the genetic association between PON1 and obesity by extending previous findings from common SNPs to include rare variants. Rare variants in *PON2* or *PON3* were not associated with obesity in this study.

Owing to our extensively characterized patient cohort, we could examine all common variants across five MASLD-related features. Remarkably, each testing group yielded at least one nominally significant result. Moreover, all nominally significant variants were located in the *PON1* gene. Together, this suggests a potential broad involvement of *PON1* in the various histological aspects of MASLD, reinforcing existing evidence connecting PON1 to liver steatosis, inflammation, fibrosis, and general MASLD [52,53,54]. A total of six unique *PON1* common variants were identified as nominally significantly related to any of the MASLD-related features. Among them, rs662 and rs854560 are well-known exonic SNPs influencing PON1 expression and activity [55,56]. rs662 is nominally significantly associated with lobular inflammation and fibrosis stage, while rs854560 is linked to the severity of MASLD. A prior study also examined the frequency of rs854560 polymorphism in MASLD and non-MASLD patients and found significant results [57]. The promotor polymorphism rs705379, also known as -108C/T, was nominally significantly associated with lobular inflammation, hepatocellular ballooning, and MASLD stage. Similar to rs662 and rs854560, rs705379 affects PON1 expression and activity [23,58]. The other promotor polymorphism rs705381, also known as -162A/G, was not associated with any MASLD-related hallmarks. The remaining variants, rs854551, rs854552, and rs3917577 are 3′UTR SNPs. Both rs854551 and rs854552 have demonstrated associations with PON1 paraoxonase activity [59], while a possible association between rs3917577 and PON1 protein expression or activity remains undescribed. Rs854551 and rs854552 both show nominal significance for association with liver fibrosis and are in LD (R^2^ = 0.785). Lastly, rs3917588 is nominally significantly associated with hepatic steatosis. Among these six SNPs, two remained significant upon FDR analysis. The first is the *PON1* promotor polymorphism, rs705379, associated with the severity of MASLD. In a previous study conducted by us, this association was also described, together with an association between this SNP, DNA methylation, and PON1 activity levels [23]. The second is the *PON1* 3′UTR SNP, rs854552, associated with the fibrosis stage. Even though many nominally significant variants did not achieve q-values below 0.05, there is a noticeable enrichment of low *p*-values in the Q-Q plots for most features (except ballooning) (Figure 3). Expansion of the cohort size could potentially aid in increasing the significance of these borderline variants. Notably, nine out of ten nominally significant and two out of two significant *PON1* SNPs have higher MAFs in the control populations (Supplementary Table S4). This indicates that contrary to the adverse metabolic effects of PON1 ablation, *PON1* SNPs might display hepatoprotective properties in various morphological hallmarks of MASLD.

Finally, the association between rare *PON* variants and MASLD-related features was examined. Although conventional research has focused mostly on common variants (through GWAS), rare variants have been reported to be associated with MASLD [60]. Contrary to common single variant tests, no associations were found for the *PON1* gene, although rare *PON1* variants have been linked to alterations in PON1 activity levels [27] and consequently, disease [61]. This negative result might be related to limited power, as we found the least amount of (very) rare variants in the *PON1* gene (Table 4). Notably, very significant associations for rare and very rare *PON2* variants with liver fibrosis were revealed. To the best of our knowledge, we are the first to report that natural genetic variation in *PON2* might be meaningful in the etiology and/or progression of MASLD-related liver fibrosis. Recent work supports this hypothesis, as (i) loss of *PON2* in an in vitro cell model of MASLD led to disruption of pathways related to MASLD pathogenesis, including hepatic fibrosis [62], (ii) exacerbated cardiac fibrosis is reported for *PON2* deficiency in a mouse transverse aortic constriction model [63] and (iii) PON2 was identified as the cellular target of vutiglabridin and necessary for alleviating MASLD-related hallmarks [64]. These functional studies, combined with our genetic results, indicate a promising foundation for further (genetic) research on PON2 in the context of MASLD, particularly focusing on fibrosis. Concomitantly, a more thorough understanding of PON2’s role in MASLD could highlight a potential significance for genetic risk profiling and personalized treatment strategies. Lastly, some (borderline) significant associations for rare *PON3* variants associated with liver fibrosis stage and lobular inflammation are described for certain burden tests. However, the evidence for this association is relatively weak, as the significance is inconsistent across burden tests.

### Advantages, Limitations, and Future Perspectives

Our study benefits from the extensively characterized patient cohort, which allows us to investigate the association between naturally occurring genetic variants and specific MASLD-related histological characteristics including hepatic steatosis, lobular inflammation, hepatocellular ballooning, and hepatic fibrosis. Identifying genes with significant associations with any of these hallmarks provides valuable insights into the possible pathophysiological mechanisms underlying MASLD. However, as some of these associations are novel, replication of our findings in an independent cohort is necessary to validate and strengthen the robustness of our results.

Given the abundance of detected variants, particularly rare variants, functional assessment of individual variants is beyond the scope of this research. Alternatively, as these (rare) genetic variants are numerous, and presumed to alter protein function, we postulate that PON expression and/or activity (encapsulating not only genetic but also environmental influences) might be an interesting target for further exploration, i.e., PON2 as a possible biomarker in patients with MASLD-associated liver fibrosis.

The relatively limited size of our cohorts poses a challenge in achieving robust statistical significance. To partially compensate for this limitation, the cumulative effect of all rare and very rare variants per gene was calculated. Despite this approach, cohort sizes may still have been too small to detect all genetic associations.

Furthermore, future exploration of possible epigenetic regulation and crosstalk between genetic *PON* variants and DNA methylation could provide deeper insights into the mechanisms contributing to clinical MASLD phenotype [23,65].

## 5. Conclusions

Our study further substantiates the relevance of *PON1* in obesity and various MASLD-related liver features, by extending previous findings from common SNPs to include rare variants. Finally, we are the first to report an association between rare *PON2* variants and MASLD-related liver fibrosis. Given the crucial role of liver fibrosis in clinical MASLD outcome prediction, PON2 might prove a promising target for future research, including exploring its potential as a biomarker or as a possible therapeutic target.

## Figures and Tables

**Table 1:** Cohort characteristics.

	Patients with Obesity (*n*= 744)	Lean Cohort (*n* = 507)
Age (years ± SD)	43.3 ± 12.6	34.8 ± 7.0
BMI (kg/m^2^ ± SD)	38.7 ± 6.2	22.0 ± 1.6
Gender (female/male)	532/212	315/192

For each cohort, the number of included individuals, mean age, body mass index (BMI) ± standard deviation (SD), and gender distribution are displayed.

**Table 2:** Extensive patient cohort characteristics.

	Mean ± SD	Minimum–Maximum
Age (years)	43.29 ± 12.57	18–74
Weight (kg)	110.86 ± 21.45	68.8–226.6
Height (m)	1.69 ± 0.09	1.47–2.06
BMI (kg/m^2^)	38.75 ± 6.22	26.16–69.13
Waist circumference (cm)	116.91 ± 14.35	83.5–193.0
Hip circumference (cm)	122.09 ± 10.29	95.0–160.0
Waist-to-hip ratio	0.96 ± 0.11	0.67–1.35
Fat free mass (kg)	56.35 ± 12.28	36.5–110.0
Fat mass (kg)	54.34 ± 15.02	22.5–134.5
Fat mass percentage	48.82 ± 7.52	26.0–65.3
Total abdominal adipose tissue (cm^2^)	800.56 ± 182.02	338–1386
Visceral abdominal tissue (cm^2^)	199.01 ± 89.70	29–567
Subcutaneous abdominal tissue (cm^2^)	601.55 ± 155.39	230–1059
Systolic blood pressure (mmHg)	127.50 ± 14.52	90–180
Diastolic blood pressure (mmHg)	75.62 ± 10.25	49–115
Creatinine kinase (mg/dL)	0.81 ± 0.18	0.38–1.64
Aspartate aminotransferase (U/L)	23.52 ± 13.27	7–133
Alanine aminotransferase (U/L)	35.36 ± 22.17	7–265
Alkaline phosphatase (U/L)	80.29 ± 22.16	30.00–236.03
Gamma-glutamyltransferase (U/L)	43.42 ± 33.75	11.79–315.16
Total cholesterol (mg/dL)	199.37 ± 39.43	75.00–400.44
High-density lipoprotein cholesterol (mg/dL)	51.03 ± 14.05	24.00–107.17
Triglycerides (mg/dL)	154.04 ± 82.82	33.04–823.01
Low-density lipoprotein cholesterol (mg/dL)	117.78 ± 34.79	17.60–294.76
HOMA-IR	4.30 ± 6.66	0.07–147.65

Mean ± SD values for baseline measurements relating to metabolic and liver health of the patients with obesity (N = 744) are represented. Lower and upper limits are displayed (minimum–maximum). Fat mass calculations are based on impedance measurements and abdominal fat tissue is measured by CT scan. BMI = body mass index, HOMA-IR = homeostatic model assessment for insulin resistance.

**Table 3:** Overview of common variants identified by single-molecule molecular inversion probes (smMIPs) sequencing of *PON1*, *PON2*, and *PON3*.

LD Block	Gene	Genomic Position (hg19)	Genetic Region	Amino Acid Change	SNP ID	Obesity		Steatosis Grade		Lobular Inflammation		Hepatocellular Ballooning		Fibrosis Stage		MASLD Stage	
						*p*-Value	*q*-Value	*p*-Value	*q*-Value	*p*-Value	*q*-Value	*p*-Value	*q*-Value	*p*-Value	*q*-Value	*p*-Value	*q*-Value
**1**	*PON1*	7:94927677A>G	3′UTR		rs854551	0.96	0.98	0.11	0.84	0.45	0.71	0.86	1.00	**0.01**	0.08	0.54	0.93
	*PON1*	7:94927924C>T	3′UTR		rs854552	0.71	0.97	0.79	0.97	0.57	0.76	0.80	1.00	**<0.001**	**0.01**	0.20	0.84
**2**	*PON1*	7:94927708T>C	3′UTR		rs3917577	1.00	0.98	**0.02**	0.52	**0.05**	0.27	0.39	1.00	0.10	0.38	0.12	0.75
**3**	*PON1*	7:94937446T>C	Exonic	p.Q192R	rs662	0.59	0.97	0.29	0.93	**0.04**	0.24	0.74	1.00	**0.03**	0.13	0.80	0.95
**4**	*PON1*	7:94946084A>T	Exonic	p.L55M	rs854560	0.12	0.91	1.00	0.98	0.13	0.42	0.49	1.00	0.67	0.80	**0.02**	0.32
**5**	*PON1*	7:94953895G>A	5′UTR		rs705379	0.93	0.98	0.11	0.84	**0.01**	0.09	**0.05**	1.00	0.06	0.26	**<0.001**	**0.01**
**6**	*PON1*	7:94953949T>C	5′UTR		rs705381	0.77	0.97	0.40	0.95	0.08	0.29	0.86	1.00	0.29	0.64	0.29	0.88
**7**	*PON3*	7:95001555C>T	Exonic	p.A99A	rs1053275	0.51	0.96	0.87	0.98	1.00	0.84	0.55	1.00	0.83	0.84	0.44	0.92
	*PON3*	7:95024046A>G	Intronic		rs11970910	0.46	0.96	0.94	0.98	0.72	0.80	0.16	1.00	0.94	0.85	0.36	0.90
**8**	*PON3*	7:95025600G>A	Exonic	p.F21F	rs13226149	0.37	0.95	0.58	0.97	0.74	0.80	0.31	1.00	0.53	0.76	0.39	0.91
**9**	*PON2*	7:95034253G>GTTA	3′UTR		rs3918	0.19	0.91	0.78	0.97	1.00	0.84	0.39	1.00	1.00	0.86	0.79	0.95
	*PON2*	7:95034775G>C	Exonic	p.S311C	rs7493	0.23	0.95	0.92	0.98	0.86	0.82	0.48	1.00	0.93	0.85	0.72	0.95
	*PON2*	7:95039445C>T	Intronic		rs2286232	0.18	0.91	0.71	0.97	0.93	0.84	0.34	1.00	0.94	0.85	0.86	0.96
	*PON2*	7:95041016G>C	Exonic	p.A148G	rs12026	0.21	0.91	0.51	0.96	0.93	0.84	0.39	1.00	0.94	0.85	0.72	0.95
**10**	*PON2*	7:95034821A>T	Intronic		rs9641164	0.84	0.98	0.26	0.93	0.31	0.63	0.93	1.00	0.93	0.85	0.70	0.95

The 15 common variants identified through smMIPs sequencing are shown. Some of these are in linkage disequilibrium (LD-block). The LD blocks one, seven, and nine have an R^2^ value of 0.79, 0.92, and 1.0, respectively. For each variant, some general descriptives are displayed along with a *p*-value (Fisher’s Exact Test) and *q*-value (FDR) for every testing group. Nominally significant results (*p* ≤ 0.05) and results with a *q*-value ≤ 0.05 are displayed in bold. The ensemble gene IDs used are ENSG00000005421.4, ENSG00000105854.8, and ENSG00000105852.6 for *PON1*, *PON2*, and *PON3*, respectively.

**Table 4:** Overview of rare and very variants identified by smMIPs sequencing.

Gene	Rare Variants (N)	Very Rare Variants (N)
*PON1*	21	17
*PON2*	21	19
*PON3*	37	25

For every gene, the amount of rare and very rare variants is displayed.

**Table 5:** Gene-based association tests.

		*PON1*		*PON2*		*PON3*	
		Rare Variants	Very Rare Variants	Rare Variants	Very Rare Variants	Rare Variants	Very Rare Variants
Obesity	C-alpha	**0.01**	0.37	**0.01**	0.14	**0.03**	0.15
	Fisher	**<0.01**	**0.04**	**0.05**	0.44	0.44	1.00
	KBAC	**<0.001**	**0.01**	0.96	0.93	0.78	0.57
	VT	**<0.01**	**0.03**	0.96	0.93	0.92	0.77
Steatosis Grade	C-alpha	0.63	0.39	0.59	0.88	0.72	0.80
	Fisher	0.61	0.36	1.00	1.00	0.38	1.00
	KBAC	0.72	0.81	0.41	0.22	0.13	0.41
	VT	0.59	0.75	0.62	0.39	0.22	0.46
Lobular Inflammation	C-alpha	0.14	0.13	0.94	0.79	0.41	0.49
	Fisher	0.82	0.58	0.72	1.00	0.07	1.00
	KBAC	0.11	0.50	0.73	0.55	**0.02**	0.29
	VT	0.08	0.61	0.56	0.37	**0.04**	0.29
Hepatocellular Ballooning	C-alpha	0.79	0.63	0.52	0.50	0.78	0.83
	Fisher	0.82	0.78	0.44	0.68	0.79	0.64
	KBAC	0.47	0.65	0.12	0.15	0.33	0.61
	VT	0.51	0.67	0.20	0.27	0.31	0.59
Fibrosis Stage	C-alpha	**0.01**	**0.05**	**<0.001**	**<0.01**	0.09	0.83
	Fisher	1.00	0.42	**<0.01**	**0.01**	0.14	1.00
	KBAC	0.34	0.67	**<0.01**	**0.01**	**0.05**	0.58
	VT	0.74	0.77	**<0.001**	**<0.01**	**0.05**	0.61
MASLD Stage	C-alpha	0.14	0.14	0.27	0.28	0.68	0.82
	Fisher	0.35	1.00	0.43	0.43	0.19	1.00
	KBAC	**0.04**	0.24	0.29	0.29	0.12	0.35
	VT	0.07	0.35	0.11	0.14	0.11	0.37

The cumulative effect of all rare (MAF < 0.01) and very rare (MAF < 0.0012) variants per gene are, respectively, analyzed across all five testing groups. Significant results (*p* ≤ 0.05) are displayed in bold.

## Data Availability

The data presented in this study are available on request from the corresponding author. The data are not publicly available due to privacy.

## Supplementary Materials

The following supporting information can be downloaded at: https://www.mdpi.com/article/10.3390/antiox13091051/s1, Table S1: Liver injury NASH-CRN scoring system; Table S2: smMIPs sequencing quality; Table S3: Genetic variants detected by smMIPs sequencing; Table S4: Common variants with allele frequencies

## References

[1] Lim G.E.H., Tang A., Ng C.H., Chin Y.H., Lim W.H., Tan D.J.H., Yong J.N., Xiao J., Lee C.W., Chan M. (2023). An Observational Data Meta-analysis on the Differences in Prevalence and Risk Factors between MAFLD vs NAFLD. Clin. Gastroenterol. Hepatol..

[2] Rinella M.E., Lazarus J.V., Ratziu V., Francque S.M., Sanyal A.J., Kanwal F., Romero D., Abdelmalek M.F., Anstee Q.M., Arab J.P. (2023). A multisociety Delphi consensus statement on new fatty liver disease nomenclature. J. Hepatol..

[3] Muthiah M.D., Cheng Han N., Sanyal A.J. (2022). A clinical overview of non-alcoholic fatty liver disease: A guide to diagnosis, the clinical features, and complications-What the non-specialist needs to know. Diabetes Obes. Metab..

[4] Younossi Z.M., Koenig A.B., Abdelatif D., Fazel Y., Henry L., Wymer M. (2016). Global epidemiology of nonalcoholic fatty liver disease-Meta-analytic assessment of prevalence, incidence, and outcomes. Hepatology.

[5] Fernández-Sánchez A., Madrigal-Santillán E., Bautista M., Esquivel-Soto J., Morales-González A., Esquivel-Chirino C., Durante-Montiel I., Sánchez-Rivera G., Valadez-Vega C., Morales-González J.A. (2011). Inflammation, oxidative stress, and obesity. Int. J. Mol. Sci..

[6] Ma Y., Lee G., Heo S.Y., Roh Y.S. (2021). Oxidative Stress Is a Key Modulator in the Development of Nonalcoholic Fatty Liver Disease. Antioxidants.

[7] Masarone M., Rosato V., Dallio M., Gravina A.G., Aglitti A., Loguercio C., Federico A., Persico M. (2018). Role of Oxidative Stress in Pathophysiology of Nonalcoholic Fatty Liver Disease. Oxid. Med. Cell Longev..

[8] Ruiz-Ojeda F.J., Olza J., Gil Á., Aguilera C.M., del Moral A.M., Aguilera García C.M. (2018). Chapter 1—Oxidative Stress and Inflammation in Obesity and Metabolic Syndrome. Obesity.

[9] Oliveira C.P., Stefano J.T. (2015). Genetic polymorphisms and oxidative stress in non-alcoholic steatohepatitis (NASH): A mini review. Clin. Res. Hepatol. Gastroenterol..

[10] Rupérez A.I., Gil A., Aguilera C.M. (2014). Genetics of oxidative stress in obesity. Int. J. Mol. Sci..

[11] Mackness M.I., Arrol S., Durrington P.N. (1991). Paraoxonase prevents accumulation of lipoperoxides in low-density lipoprotein. FEBS Lett..

[12] Mackness M.I., Arrol S., Mackness B., Durrington P.N. (1997). Alloenzymes of paraoxonase and effectiveness of high-density lipoproteins in protecting low-density lipoprotein against lipid peroxidation. Lancet.

[13] Reddy S.T., Wadleigh D.J., Grijalva V., Ng C., Hama S., Gangopadhyay A., Shih D.M., Lusis A.J., Navab M., Fogelman A.M. (2001). Human paraoxonase-3 is an HDL-associated enzyme with biological activity similar to paraoxonase-1 protein but is not regulated by oxidized lipids. Arterioscler. Thromb. Vasc. Biol..

[14] Brancale J., Vilarinho S. (2021). A single cell gene expression atlas of 28 human livers. J. Hepatol..

[15] Guilliams M., Bonnardel J., Haest B., Vanderborght B., Wagner C., Remmerie A., Bujko A., Martens L., Thoné T., Browaeys R. (2022). Spatial proteogenomics reveals distinct and evolutionarily conserved hepatic macrophage niches. Cell.

[16] Devarajan A., Bourquard N., Hama S., Navab M., Grijalva V.R., Morvardi S., Clarke C.F., Vergnes L., Reue K., Teiber J.F. (2011). Paraoxonase 2 deficiency alters mitochondrial function and exacerbates the development of atherosclerosis. Antioxid. Redox Signal.

[17] Higgins G.C., Beart P.M., Shin Y.S., Chen M.J., Cheung N.S., Nagley P. (2010). Oxidative stress: Emerging mitochondrial and cellular themes and variations in neuronal injury. J. Alzheimers Dis..

[18] Horke S., Witte I., Wilgenbus P., Krüger M., Strand D., Förstermann U. (2007). Paraoxonase-2 reduces oxidative stress in vascular cells and decreases endoplasmic reticulum stress-induced caspase activation. Circulation.

[19] Camps J., Marsillach J., Joven J. (2009). The paraoxonases: Role in human diseases and methodological difficulties in measurement. Crit. Rev. Clin. Lab. Sci..

[20] Ng C.J., Wadleigh D.J., Gangopadhyay A., Hama S., Grijalva V.R., Navab M., Fogelman A.M., Reddy S.T. (2001). Paraoxonase-2 is a ubiquitously expressed protein with antioxidant properties and is capable of preventing cell-mediated oxidative modification of low density lipoprotein. J. Biol. Chem..

[21] Priyanka K., Singh S., Gill K. (2019). Paraoxonase 3: Structure and Its Role in Pathophysiology of Coronary Artery Disease. Biomolecules.

[22] Meneses M.J., Silvestre R., Sousa-Lima I., Macedo M.P. (2019). Paraoxonase-1 as a Regulator of Glucose and Lipid Homeostasis: Impact on the Onset and Progression of Metabolic Disorders. Int. J. Mol. Sci..

[23] Diels S., Cuypers B., Tvarijonaviciute A., Derudas B., Van Dijck E., Verrijken A., Van Gaal L.F., Laukens K., Lefebvre P., Ceron J.J. (2021). A targeted multi-omics approach reveals paraoxonase-1 as a determinant of obesity-associated fatty liver disease. Clin. Epigenet..

[24] Dasgupta S., Demirci F.Y., Dressen A.S., Kao A.H., Rhew E.Y., Ramsey-Goldman R., Manzi S., Kammerer C.M., Kamboh M.I. (2011). Association analysis of PON2 genetic variants with serum paraoxonase activity and systemic lupus erythematosus. BMC Med. Genet..

[25] Riedmaier S., Klein K., Winter S., Hofmann U., Schwab M., Zanger U.M. (2011). Paraoxonase (PON1 and PON3) Polymorphisms: Impact on Liver Expression and Atorvastatin-Lactone Hydrolysis. Front. Pharmacol..

[26] Cirulli E.T., Goldstein D.B. (2010). Uncovering the roles of rare variants in common disease through whole-genome sequencing. Nat. Rev. Genet..

[27] Kim D.S., Burt A.A., Crosslin D.R., Robertson P.D., Ranchalis J.E., Boyko E.J., Nickerson D.A., Furlong C.E., Jarvik G.P. (2013). Novel common and rare genetic determinants of paraoxonase activity: FTO, SERPINA12, and ITGAL. J. Lipid Res..

[28] Francque S.M.A., Verrijken A., Mertens I., Hubens G., Van Marck E., Pelckmans P., Michielsen P., Van Gaal L. (2012). Noninvasive Assessment of Nonalcoholic Fatty Liver Disease in Obese or Overweight Patients. Clin. Gastroenterol. Hepatol..

[29] Kleiner D.E., Brunt E.M., Van Natta M., Behling C., Contos M.J., Cummings O.W., Ferrell L.D., Liu Y.-C., Torbenson M.S., Unalp-Arida A. (2005). Design and validation of a histological scoring system for nonalcoholic fatty liver disease. Hepatology.

[30] Pai R.K. (2019). NAFLD Histology: A Critical Review and Comparison of Scoring Systems. Curr. Hepatol. Rep..

[31] Boyle E.A., O’Roak B.J., Martin B.K., Kumar A., Shendure J. (2014). MIPgen: Optimized modeling and design of molecular inversion probes for targeted resequencing. Bioinformatics.

[32] O’Roak B.J., Vives L., Fu W., Egertson J.D., Stanaway I.B., Phelps I.G., Carvill G., Kumar A., Lee C., Ankenman K. (2012). Multiplex targeted sequencing identifies recurrently mutated genes in autism spectrum disorders. Science.

[33] Dutta D., Gagliano Taliun S.A., Weinstock J.S., Zawistowski M., Sidore C., Fritsche L.G., Cucca F., Schlessinger D., Abecasis G.R., Brummett C.M. (2019). Meta-MultiSKAT: Multiple phenotype meta-analysis for region-based association test. Genet. Epidemiol..

[34] Li H., Durbin R. (2009). Fast and accurate short read alignment with Burrows-Wheeler transform. Bioinformatics.

[35] McKenna A., Hanna M., Banks E., Sivachenko A., Cibulskis K., Kernytsky A., Garimella K., Altshuler D., Gabriel S., Daly M. (2010). The Genome Analysis Toolkit: A MapReduce framework for analyzing next-generation DNA sequencing data. Genome Res..

[36] Karczewski K.J., Francioli L.C., Tiao G., Cummings B.B., Alföldi J., Wang Q., Collins R.L., Laricchia K.M., Ganna A., Birnbaum D.P. (2020). The mutational constraint spectrum quantified from variation in 141,456 humans. Nature.

[37] Untergasser A., Cutcutache I., Koressaar T., Ye J., Faircloth B.C., Remm M., Rozen S.G. (2012). Primer3—New capabilities and interfaces. Nucleic Acids Res..

[38] San Lucas F.A., Wang G., Scheet P., Peng B. (2012). Integrated annotation and analysis of genetic variants from next-generation sequencing studies with variant tools. Bioinformatics.

[39] Storey J.D., Tibshirani R. (2003). Statistical significance for genomewide studies. Proc. Natl. Acad. Sci. USA.

[40] Lee S., Abecasis G.R., Boehnke M., Lin X. (2014). Rare-variant association analysis: Study designs and statistical tests. Am. J. Hum. Genet..

[41] Price A.L., Kryukov G.V., de Bakker P.I., Purcell S.M., Staples J., Wei L.J., Sunyaev S.R. (2010). Pooled association tests for rare variants in exon-resequencing studies. Am. J. Hum. Genet..

[42] Liu D.J., Leal S.M. (2010). A novel adaptive method for the analysis of next-generation sequencing data to detect complex trait associations with rare variants due to gene main effects and interactions. PLoS Genet..

[43] Mather K. (1945). Statistical Methods for Research Workers. Nature.

[44] Neale B.M., Rivas M.A., Voight B.F., Altshuler D., Devlin B., Orho-Melander M., Kathiresan S., Purcell S.M., Roeder K., Daly M.J. (2011). Testing for an unusual distribution of rare variants. PLoS Genet..

[45] Huen K., Harley K., Beckman K., Eskenazi B., Holland N. (2013). Associations of PON1 and genetic ancestry with obesity in early childhood. PLoS ONE.

[46] Ruperez A., López-Guarnido O., Gil F., Olza J., Gil-Campos M., Leis R., Tojo R., Cañete R., Gil Á., Aguilera C. (2013). Paraoxonase 1 activities and genetic variation in childhood obesity. Br. J. Nutr..

[47] Veiga L., Silva-Nunes J., Melão A., Oliveira A., Duarte L., Brito M. (2011). Q192R polymorphism of the paraoxonase-1 gene as a risk factor for obesity in Portuguese women. Eur. J. Endocrinol..

[48] Shih D.M., Xia Y.R., Wang X.P., Wang S.S., Bourquard N., Fogelman A.M., Lusis A.J., Reddy S.T. (2007). Decreased obesity and atherosclerosis in human paraoxonase 3 transgenic mice. Circ. Res..

[49] Shih D.M., Yu J.M., Vergnes L., Dali-Youcef N., Champion M.D., Devarajan A., Zhang P., Castellani L.W., Brindley D.N., Jamey C. (2015). PON3 knockout mice are susceptible to obesity, gallstone formation, and atherosclerosis. FASEB J.

[50] Ng C.J., Bourquard N., Grijalva V., Hama S., Shih D.M., Navab M., Fogelman A.M., Lusis A.J., Young S., Reddy S.T. (2006). Paraoxonase-2 deficiency aggravates atherosclerosis in mice despite lower apolipoprotein-B-containing lipoproteins: Anti-atherogenic role for paraoxonase-2. J. Biol. Chem..

[51] Shih D.M., Meng Y., Sallam T., Vergnes L., Shu M.L., Reue K., Tontonoz P., Fogelman A.M., Lusis A.J., Reddy S.T. (2019). PON2 Deficiency Leads to Increased Susceptibility to Diet-Induced Obesity. Antioxidants.

[52] Atamer A., Bilici A., Yenice N., Selek S., Ilhan N., Atamer Y. (2008). The importance of paraoxonase 1 activity, nitric oxide and lipid peroxidation in hepatosteatosis. J. Int. Med. Res..

[53] García-Heredia A., Kensicki E., Mohney R.P., Rull A., Triguero I., Marsillach J., Tormos C., Mackness B., Mackness M., Shih D.M. (2013). Paraoxonase-1 deficiency is associated with severe liver steatosis in mice fed a high-fat high-cholesterol diet: A metabolomic approach. J. Proteome Res..

[54] Marsillach J., Camps J., Ferré N., Beltran R., Rull A., Mackness B., Mackness M., Joven J. (2009). Paraoxonase-1 is related to inflammation, fibrosis and PPAR delta in experimental liver disease. BMC Gastroenterol..

[55] Garin M.C., James R.W., Dussoix P., Blanché H., Passa P., Froguel P., Ruiz J. (1997). Paraoxonase polymorphism Met-Leu54 is associated with modified serum concentrations of the enzyme. A possible link between the paraoxonase gene and increased risk of cardiovascular disease in diabetes. J. Clin. Investig..

[56] Humbert R., Adler D.A., Disteche C.M., Hassett C., Omiecinski C.J., Furlong C.E. (1993). The molecular basis of the human serum paraoxonase activity polymorphism. Nat. Genet..

[57] Milaciu M.V., Vesa Ș.C., Bocșan I.C., Ciumărnean L., Sâmpelean D., Negrean V., Pop R.M., Matei D.M., Pașca S., Răchișan A.L. (2019). Paraoxonase-1 Serum Concentration and PON1 Gene Polymorphisms: Relationship with Non-Alcoholic Fatty Liver Disease. J. Clin. Med..

[58] Brophy V.H., Hastings M.D., Clendenning J.B., Richter R.J., Jarvik G.P., Furlong C.E. (2001). Polymorphisms in the human paraoxonase (PON1) promoter. Pharmacogenetics.

[59] Huen K., Barcellos L., Beckman K., Rose S., Eskenazi B., Holland N. (2011). Effects of PON polymorphisms and haplotypes on molecular phenotype in Mexican-American mothers and children. Environ. Mol. Mutagen..

[60] Boonvisut S., Yoshida K., Nakayama K., Watanabe K., Miyashita H., Iwamoto S. (2017). Identification of deleterious rare variants in MTTP, PNPLA3, and TM6SF2 in Japanese males and association studies with NAFLD. Lipids Health Dis..

[61] Kim D.S., Crosslin D.R., Auer P.L., Suzuki S.M., Marsillach J., Burt A.A., Gordon A.S., Meschia J.F., Nalls M.A., Worrall B.B. (2014). Rare coding variation in paraoxonase-1 is associated with ischemic stroke in the NHLBI Exome Sequencing Project. J. Lipid Res..

[62] Shin G.C., Lee H.M., Kim N., Yoo S.K., Park H.S., Choi L.S., Kim K.P., Lee A.R., Seo S.U., Kim K.H. (2022). Paraoxonase-2 contributes to promoting lipid metabolism and mitochondrial function via autophagy activation. Sci. Rep..

[63] Li W., Kennedy D., Shao Z., Wang X., Kamdar A.K., Weber M., Mislick K., Kiefer K., Morales R., Agatisa-Boyle B. (2018). Paraoxonase 2 prevents the development of heart failure. Free Radic. Biol. Med..

[64] Shin G.C., Lee H.M., Kim N., Hur J., Yoo S.K., Park Y.S., Park H.S., Ryu D., Park M.H., Park J.H. (2024). Paraoxonase-2 agonist vutiglabridin promotes autophagy activation and mitochondrial function to alleviate non-alcoholic steatohepatitis. Br. J. Pharmacol..

[65] Declerck K., Remy S., Wohlfahrt-Veje C., Main K.M., Van Camp G., Schoeters G., Vanden Berghe W., Andersen H.R. (2017). Interaction between prenatal pesticide exposure and a common polymorphism in the PON1 gene on DNA methylation in genes associated with cardio-metabolic disease risk-an exploratory study. Clin. Epigenet..

