# Supplementary material for: A Case–Control Study Supports Genetic Contribution of the PON Gene Family in Obesity and Metabolic Dysfunction Associated Steatotic Liver Disease
Source: Antioxidants (Basel). 2024 Aug 29;13(9):1051. doi: 10.3390/antiox13091051 (PMC11440101; doi:10.3390/antiox13091051)
Supplement: Supplementary file 1 [file antioxidants-13-01051-s001.zip › antioxidants-3169066-supplementary.pdf]

Supplementary data

PPV and NPV calculations

Sanger sequencing was employed to enhance quality parameter thresholds used for variant calling in the next-generation sequencing (NGS) data, aiming to minimize both false positive and false negative variant calls. A total of 85 samples, encompassing 23,780 base pairs, were sequenced using this gold standard, resulting in the identification of 109 genetic variants. These samples and variants had also been previously targeted by single molecule Molecular Inversion Probes (smMIPs) analysis. Quality parameter thresholds for "Quality by Depth", "Total Allele Depth" and "Allelic Ratio" were fine-tuned based on key metrics, including positive predictive value (PPV) and negative predictive value (NPV). The latter are calculated from the fraction of confirmed variants from Sanger sequencing compared to all variants observed in smMIPs data (referred to as true positives) and the fraction of wild-type alleles from Sanger sequencing compared to all wild-types observed in smMIPs data (referred to as true negatives) respectively. Following the application of the three quality parameter thresholds outlined below, both positive predictive value (PPV) and negative predictive value (NPV) reached 100%.

Table A. Raw sequencing data table from smMIPs and Sanger sequencing results

		**smMIPs**		
		**R/R**	**R/A or A/A**	Total
**Sanger sequencing**	**R/R**	23615	56	23671
	**R/A or A/A**	2	109	111
	Total	23617	165	

Depiction of the sequencing data obtained from smMIPs compared to Sanger sequencing. In the smMIPs data, 2 variants are wrongly denoted as homozygous reference alleles. A total of 56 samples are wrongly labeled as being heterozygous or homozygous alternative, as they were found to be homozygous reference in the Sanger sequencing results. PPV = 109/165 = 64%, NPV = 23615/23617 = 99.99%

Criterium 1: Quality by Depth > 3.89

Table B. Sequencing data overview after application of the first criterium

		**smMIPs**		
		**R/R**	**R/A or A/A**	Total
**Sanger sequencing**	**R/R**	23615	8	23623
	**R/A or A/A**	2	108	110
	Total	23617	116	

PPV = 108/116 = 93%, NPV = 23615/23617 = 99.99%

Criterium 2: Total Allele Depth ≥ 5

Table C. Sequencing data overview after application of the first and second criterium

		**smMIPs**		
		**R/R**	**R/A or A/A**	Total
**Sanger sequencing**	**R/R**	23615	8	23623
	**R/A or A/A**	1	107	108
	Total	23616	115	

PPV = 107/115 = 93%, NPV = 23615/23616 = 99.99%

Criterium 3: Allelic Ratio: reclassify variants based on the fraction of their alternative allele(s)

Alternative allelic fraction below 20%, between 20-80% and above 80% result in homozygous reference, heterozygous and homozygous alternative variant calls respectively.

Table D. Sequencing data overview after application of the first, second and third criterium

		**smMIPs**		
		**R/R**	**R/A or A/A**	Total
**Sanger sequencing**	**R/R**	23615	0	23615
	**R/A or A/A**	0	107	107
	Total	23615	107	

PPV: 100%, NPV: 100%

1. Supplementary tables

Supplementary Table S1. Liver injury NASH-CRN scoring system

**Liver injury parameter**	**NASH-CRN Scoring System**	**Patients (*n*)**
**Steatosis Grade (S)**	0= < 5%	103
	1= 5%-33%	144
	2= 33%-66%	109
	3= >66%	77
**Lobular Inflammation (I)**	0= no foci	141
	1= <2 foci per 200× field	196
	2= 2-4 foci per 200× field	67
	3= >4 foci per 200× field	29
**Hepatocellular Ballooning (B)**	0= none	132
	1= few balloon cells	174
	2= many cells/prominent ballooning	127
**Fibrosis Stage (F)**	0= none	266
	1= perisinusoidal or periportal	86
	2= perisinusoidal and portal/periportal	50
	3= bridging fibrosis	27
	4= cirrhosis	3
**MASLD Stage**	1= No MASLD (S, B, I and F = 0)	56
	2= hepatic steatosis (S ≥ 1 (B or I can also be ≥ 1))	78
	3= MASH with mild fibrosis (S ≥ 1, I ≥ 1, B ≥ 1, F ≥ 1)	192
	4= MASH with moderate to severe fibrosis (S ≥ 1, I ≥ 1, B ≥ 1, F>1)	51

Overview of the liver injury parameters (i) steatosis grade, (ii) lobular inflammation, (iii) hepatocellular ballooning and (iv) fibrosis stage and their grading according to the NASH-CRN scoring system. The different MASLD stages are defined based on the grades of the four liver injury parameters. For each subgroup, the number of patients is displayed.

Supplementary Table S2. smMIPs sequencing quality

Cohort	Gene	Transcript	Median transcript coverage	Average transcript coverage (%)	95% CI for average transcript coverage (%)
**Control**	*PON1*	NM_000446.5	414×	99.5	99.1-99.8
	PON2	NM_000305.2	212×	99.2	98.7-99.6
	PON2	NM_001018161.1	234×	99.2	98.8-99.6
	PON3	NM_000940.2	333×	99.5	99.2-99.9
**Patient**	*PON1*	NM_000446.5	178×	99.6	99.4-99.7
	PON2	NM_000305.2	178×	99.1	99.0-99.3
	PON2	NM_001018161.1	184×	99.2	99.0-99.3
	PON3	NM_000940.2	146×	99.7	99.5-99.8

Overview of the smMIPs sequencing quality including the median transcript coverage, average transcript coverage and it's 95% confidence interval (CI) per gene transcript. Average transcript coverage and the CI are determined at a minimum coverage of 5×.

Supplementary Table S3. Genetic variants detected by smMIPs sequencing

Genomic position (hg19)	Mutation type	Genetic region	Gene	MAF control	MAF case
**7:94927677A>G**	.	3'UTR	*PON1*	0.196573	0.197609
**7:94927703A>T**	.	3'UTR	*PON1*	0.004024	0.002813
**7:94927708T>C**	.	3'UTR	*PON1*	0.097586	0.098453
**7:94927734TA>T**	.	3'UTR	*PON1*	0.002012	0.012658
**7:94927865C>T**	.	3'UTR	*PON1*	0.004024	0.005634
**7:94927924C>T**	.	3'UTR	*PON1*	0.265060	0.257746
**7:94928081C>CA**	.	3'UTR	*PON1*	0.001029	0.002845
**7:94928120CAG>C**	.	3'UTR	*PON1*	0.000000	0.000709
**7:94928328T>G**	Silent T332	exonic	*PON1*	0.000000	0.001406
**7:94931659A>C**	.	intronic	*PON1*	0.001004	0.000000
**7:94931707A>G**	.	intronic	*PON1*	0.000000	0.000703
**7:94935589C>T**	.	intronic	*PON1*	0.000000	0.000703
**7:94937412C>T**	Silent S203	exonic	*PON1*	0.001002	0.000000
**7:94937418C>T**	Silent A201	exonic	*PON1*	0.000000	0.002119
**7:94937419G>A**	Missense A201V	exonic	*PON1*	0.001002	0.003531
**7:94937446T>C**	Missense Q192R	exonic	*PON1*	0.284569	0.295198
**7:94937528A>G**	.	intronic	*PON1*	0.000000	0.000703
**7:94940880A>C**	Missense M127R	exonic	*PON1*	0.000000	0.002116
**7:94944735A>G**	Missense L90P	exonic	*PON1*	0.001004	0.000000
**7:94944826A>G**	.	intronic	*PON1*	0.000000	0.000704
**7:94946084A>T**	Missense L55M	exonic	*PON1*	0.356137	0.387482
**7:94947671C>T**	Missense V37I	exonic	*PON1*	0.001020	0.000000
**7:94947769G>T**	.	intronic	*PON1*	0.000000	0.000797
**7:94953733T>C**	Missense N19D	exonic	*PON1*	0.002008	0.003536
**7:94953771G>A**	Missense A6V	exonic	*PON1*	0.000000	0.000706

**7:94953849G>T**	.	5′UTR	*PON1*	0.001002	0.000000
**7:94953895G>A**	.	5′UTR	*PON1*	0.486948	0.485190
**7:94953913G>C**	.	5′UTR	*PON1*	0.044792	0.053161
**7:94953949T>C**	.	5′UTR	*PON1*	0.258873	0.252874
**7:94989168A>T**	.	downstream	*PON3*	0.000000	0.000971
**7:94989173A>C**	.	downstream	*PON3*	0.000000	0.000846
**7:94989270T>C**	.	3′UTR	*PON3*	0.001012	0.000000
**7:94989297G>A**	Silent Y351	exonic	*PON3*	0.000000	0.000704
**7:94989379C>G**	Missense G324A	exonic	*PON3*	0.001018	0.000000
**7:94989379C>T**	Missense G324D	exonic	*PON3*	0.000000	0.001351
**7:94993163A>G**	.	intronic	*PON3*	0.001002	0.000000
**7:94993238T>A**	Missense E211V	exonic	*PON3*	0.000000	0.000703
**7:94993261A>G**	Silent Y203	exonic	*PON3*	0.002008	0.004923
**7:94993314T>A**	Missense T186S	exonic	*PON3*	0.001006	0.000000
**7:94993387G>A**	.	intronic	*PON3*	0.002024	0.000000
**7:94996678T>C**	Missense K164E	exonic	*PON3*	0.001018	0.000000
**7:94996732C>T**	Missense E146K	exonic	*PON3*	0.000000	0.000703
**7:94996760C>A**	Missense M136I	exonic	*PON3*	0.001006	0.000000
**7:94996790C>T**	Silent V126	exonic	*PON3*	0.004024	0.000000
**7:95001458C>T**	.	intronic	*PON3*	0.000000	0.001410
**7:95001464C>T**	.	intronic	*PON3*	0.002008	0.002116
**7:95001555C>T**	Silent A99	exonic	*PON3*	0.477912	0.492243
**7:95001590T>C**	Missense M88V	exonic	*PON3*	0.000000	0.001435
**7:95006801G>A**	.	intronic	*PON3*	0.005030	0.000000
**7:95006811T>A**	.	intronic	*PON3*	0.005030	0.000000
**7:95006855C>T**	.	intronic	*PON3*	0.005030	0.000000
**7:95006856G>A**	.	intronic	*PON3*	0.002012	0.001445
**7:95006859T>A**	.	intronic	*PON3*	0.000000	0.000722
**7:95006911G>A**	.	intronic	*PON3*	0.007042	0.001416
**7:95006926C>G**	.	intronic	*PON3*	0.000000	0.001416
**7:95006931G>A**	.	intronic	*PON3*	0.001006	0.000000
**7:95006939G>C**	.	intronic	*PON3*	0.041247	0.021246
**7:95006998G>A**	.	intronic	*PON3*	0.000000	0.000749
**7:95019448A>C**	.	intronic	*PON3*	0.001010	0.000703
**7:95019465C>T**	.	splicing	*PON3*	0.001010	0.000000
**7:95019575C>T**	.	intronic	*PON3*	0.002183	0.003771
**7:95023931C>T**	.	intronic	*PON3*	0.000000	0.000744
**7:95024006C>A**	Missense R32L	exonic	*PON3*	0.001008	0.000000
**7:95024007G>A**	Stopgain R32*	exonic	*PON3*	0.004032	0.002817
**7:95024046A>G**	.	intronic	*PON3*	0.467677	0.483803
**7:95025547A>G**	.	intronic	*PON3*	0.001022	0.000000
**7:95025553AAG>A**	.	intronic	*PON3*	0.006135	0.005714
**7:95025600G>A**	Silent F21	exonic	*PON3*	0.260121	0.276989
**7:95025658C>T**	Missense G2E	exonic	*PON3*	0.001018	0.000000
**7:95025673T>G**	.	5′UTR	*PON3*	0.001020	0.000000
**7:95025693G>A**	.	5′UTR	*PON3*	0.012245	0.012251

**7:95034207C>T**	.	3’UTR	*PON2*	0.000000	0.001416
**7:95034252G>GTTA**	.	3’UTR	*PON2*	0.226815	0.250706
**7:95034407T>C**	.	3’UTR	*PON2*	0.001002	0.000000
**7:95034654A>G**	Silent Y351	exonic	*PON2*	0.001002	0.000000
**7:95034775G>C**	Missense S311C	exonic	*PON2*	0.228457	0.250000
**7:95034821A>T**	.	intronic	*PON2*	0.205411	0.201289
**7:95035376T>C**	.	intronic	*PON2*	0.013889	0.011268
**7:95035508C>T**	Missense G277R	exonic	*PON2*	0.001018	0.000000
**7:95035582A>T**	.	intronic	*PON2*	0.194898	0.243662
**7:95036368A>C**	Silent V236	exonic	*PON2*	0.000000	0.000703
**7:95039222G>A**	Missense P229L	exonic	*PON2*	0.001004	0.000000
**7:95039394C>G**	Missense V172L	exonic	*PON2*	0.001006	0.000000
**7:95039445C>T**	.	intronic	*PON2*	0.225806	0.250352
**7:95039485A>C**	.	intronic	*PON2*	0.001010	0.000703
**7:95040988T>C**	silent T157	exonic	*PON2*	0.001014	0.000703
**7:95040996G>A**	Silent L155	exonic	*PON2*	0.001014	0.000000
**7:95041016G>C**	Missense A148G	exonic	*PON2*	0.227642	0.250352
**7:95041135C>T**	.	intronic	*PON2*	0.003119	0.000734
**7:95041699G>A**	missense R98W	exonic	*PON2*	0.000000	0.000703
**7:95041704CT>C**	frameshift deletion p.Arg96GlyfsTer5	exonic	*PON2*	0.001002	0.002110
**7:95045643C>A**	.	intronic	*PON2*	0.001027	0.000000
**7:95064133C>T**	.	intronic	*PON2*	0.001006	0.001466
**7:95064147G>A**	.	intronic	*PON2*	0.000000	0.000733
**7:95064160C>G**	.	intronic	*PON2*	0.001006	0.000000
**7:95064166G>A**	.	intronic	*PON2*	0.008048	0.001466
**7:95064268C>T**	.	5’UTR	*PON2*	0.021008	0.028942
**7:95064294C>T**	.	5’UTR	*PON2*	0.001027	0.000769
**7:95064315G>C**	.	5’UTR	*PON2*	0.001033	0.000835
**7:95064362C>A**	.	5’UTR	*PON2*	0.000000	0.000756

Overview of all the variants in *PON1*, *PON2* and *PON3* detected by smMIPs. Genetic position (hg19), mutation type (including amino acid changes), genetic region, gene, and minor allele frequencies (MAF) are displayed in the lean control population (N=501) and in the population with obesity (N=741). The ensemble gene IDs used are ENSG00000005421.4, ENSG00000105854.8 and ENSG00000105852.6 for *PON1*, *PON2* and *PON3* respectively.

Supplementary Table S4. Common variants with allele frequencies

LD block	Gene	SNP ID	Obesity		Steatosis Grade		Lobular Inflammation		Hepatocellular Ballooning		Fibrosis Stage		MASLD Stage		MAF NFE
			**contr**	**case**	**contr**	**case**	**contr**	**case**	**contr**	**case**	**contr**	**case**	**contr**	**case**	
**1**	*PON1*	rs854551	797/992	1141/1422	162/202	491/660	217/280	436/582	200/262	453/600	**438/528**	**251/332**	205/268	361/486	0,80
	PON1	rs854552	732/996	1054/1420	148/202	475/660	206/280	417/582	191/262	432/600	**<u>409/528</u>**	**<u>222/332</u>**	202/268	344/486	0,74
**2**	*PON1*	rs3917577	97/994	140/1422	**29/202**	**54/660**	**36/280**	**50/582**	30/262	56/600	60/528	26/332	31/268	39/486	0,09
**3**	*PON1*	rs662	284/998	418/1416	65/202	187/660	**95/280**	**157/582**	79/262	173/600	**139/524**	**112/332**	78/268	136/486	0,29
**4**	*PON1*	rs854560	354/994	551/1422	76/202	247/660	115/280	208/582	103/262	220/600	210/528	127/332	**117/268**	**169/486**	0,36
**5**	*PON1*	rs705379	485/996	688/1418	107/202	303/656	**153/280**	**257/578**	**139/262**	**271/596**	269/526	147/332	**<u>152/268</u>**	**<u>207/482</u>**	0,48
**6**	*PON1*	rs705381	710/958	1040/1392	147/192	473/644	211/270	409/566	191/256	429/580	378/512	256/332	198/260	342/472	0,75
**7**	*PON3*	rs1053275	476/996	698/1418	89/202	296/658	125/280	260/580	113/262	272/598	257/526	165/332	114/268	220/484	0,47
	PON3	rs11970910	463/990	687/1420	96/202	311/660	135/280	272/582	114/262	293/600	253/528	160/332	118/268	231/486	0,46
**8**	*PON3*	rs13226149	257/988	390/1408	50/202	177/656	72/280	155/578	63/262	164/596	136/520	93/330	65/268	132/482	0,27
**9**	*PON2*	rs3918	225/992	355/1416	48/202	164/660	69/280	143/582	59/262	153/600	130/524	83/332	63/268	119/486	0,23
	PON2	rs7493	228/998	349/1396	46/200	155/654	64/278	137/576	57/260	144/594	126/516	81/326	59/266	113/480	0,24
	PON2	rs2286232	224/992	356/1422	47/202	163/660	69/280	141/582	58/262	152/600	130/528	83/332	63/268	118/486	0,24
	PON2	rs12026	224/984	356/1422	45/202	164/660	67/280	142/582	58/262	151/600	130/528	83/332	62/268	119/486	0,24
**10**	*PON2*	rs9641164	205/998	281/1396	45/196	126/658	61/276	110/578	51/258	120/596	107/516	66/326	48/264	94/484	0,19

Overview of the common variants and the frequency of the sequenced alleles. All ratios represent the amount of alternative alleles compared to the total number of alleles genotyped. Tests with nominal significance are indicated in bold. Tests with false discovery rate (FDR) below 0.05 are indicated in bold and underlined. "contr" is short for controls and represents the control population which is lean individuals, no hepatic steatosis, no lobular inflammation, no hepatocellular ballooning, no fibrosis and no MASLD or only hepatic steatosis respectively. Cases represent patients with obesity, hepatic steatosis, lobular inflammation, hepatocellular ballooning, fibrosis and MASH with varying degrees of fibrosis respectively. In the final column the minor allele frequency of non-Finnish European ethnicity is displayed, as recorded in gnomAD (v4.0.0.)
